# *Lespedeza bicolor* Turcz. Honey Prevents Inflammation Response and Inhibits Ferroptosis by *Nrf2*/*HO-1* Pathway in DSS-Induced Human Caco-2 Cells

**DOI:** 10.3390/antiox13080900

**Published:** 2024-07-25

**Authors:** Caijun Ren, Yuying Zhu, Qiangqiang Li, Miao Wang, Suzhen Qi, Dandan Sun, Liming Wu, Liuwei Zhao

**Affiliations:** 1 State Key Laboratory of Resource Insects, Institute of Apicultural Research, Chinese Academy of Agricultural Sciences, Beijing 100093, China; 82101221123@caas.cn (C.R.); 82101235558@caas.cn (Y.Z.); liqiangqiang@caas.cn (Q.L.); wangmiao03@caas.cn (M.W.); qisuzhen@caas.cn (S.Q.); sundandan@caas.cn (D.S.); 2 Risk Assessment Laboratory for Bee Products Quality and Safety of Ministry of Agriculture, Beijing 100093, China

**Keywords:** *Lespedeza bicolor* Turcz. honey, Trifolin, anti-inflammatory activity, ferroptosis, *Nrf2*/*HO-1* pathway

## Abstract

*Lespedeza bicolor* Turcz. (*L. bicolor*) honey, a monofloral honey, has garnered increased attention due to its origin in the *L. bicolor* plant. A previous study has shown that *L. bicolor* honey can ameliorate inflammation. In this study, we aimed to investigate the effects of *L. bicolor* honey extract and its biomarker (Trifolin) on DSS-induced ulcerative colitis (UC). Our results demonstrated that *L. bicolor* honey extract and Trifolin significantly increased the expression levels of the tight junction cytokines *Claudin-1* and *ZO-1*. Additionally, they decreased the pro-inflammatory factors *TNF-α* and *IL-6* and enhanced the antioxidant factors *NQO1* and *GSTA1*. Based on metabolomic analyses, *L. bicolor* honey extract and Trifolin regulated the progression of UC by inhibiting ferroptosis. Mechanistically, they improved the levels of SOD and iron load, increased the GSH/GSSG ratio, reduced MDA content and ROS release, and upregulated the *Nrf2*/*HO-1* pathway, thereby inhibiting DSS-induced UC. Moreover, the expression levels of ferroptosis-related genes indicated that they decreased *FTL*, *ACSL4*, and *PTGS2* while increasing *SLC7A11* expression to resist ferroptosis. In conclusion, our study found that *L. bicolor* honey improves DSS-induced UC by inhibiting ferroptosis by activating the *Nrf2*/*HO-1* pathway. These findings further elucidate the understanding of anti-inflammatory and antioxidant activities of *L. bicolor* honey.

## 1. Introduction

Honey is a sweet substance collected by bees from plant nectar, secretions, or insect secretions, which they store and fully brew into the hive [1]. It contains various nutrients, including different types of sugars, proteins, organic acids, and phenolic compounds [2]. Consequently, many types of honey have been used to treat diseases such as IBD, atherosclerosis, stomatitis, and sinusitis [3,4,5,6].

In recent years, monofloral honey has gained significant attention from consumers due to its unique nutritional properties. *Lespedeza bicolor* Turcz. (*L. bicolor*) honey, derived from the *L. bicolor* plant, is considered a typical medicinal honey. This plant is known for its functions in clearing heat, detoxifying, promoting blood circulation, removing blood stasis, moisturizing the lungs, and relieving cough [7]. According to our previous study, *L. bicolor* honey exhibited rich in phenolic active ingredients (such as chlorogenic acid, ferulic acid, vitexin, rutin, gallic acid, myricitrin, morin, trifolin, glycitein, wogonin, butin and liquiritigenin) for playing anti-inflammatory activity by regulating sphingolipid metabolism and necroptosis pathway in an LPS-induced mouse macrophage RAW 264.7 cell model [8]. Additionally, several studies have found that adding wildflower honey to oral rehydration solutions can effectively improve the symptoms of gastroenteritis in children under five years old [9]. Through in vitro intestinal simulations using human fecal microbiota, wildflower honey has been shown to regulate intestinal inflammation by increasing the abundance of beneficial *Lactobacillus*, reducing the abundance of harmful Gram-negative bacteria, decreasing the production of short-chain fatty acids, and inhibiting the expression of pro-inflammatory factors [10]. These studies indicate that specific honey is effective in preventing and treating human inflammatory diseases.

Ulcerative colitis (UC) is a chronic inflammatory disease of the gastrointestinal tract that primarily affects the colon [11]. The exact cause of UC remains unclear, but it often presents with symptoms such as abdominal pain and diarrhea [11]. UC is challenging to cure and carries the risk of progressing to cancer [12]. At present, Caco-2 cells induced by DSS are widely used as a cell model for exploring UC. It has been reported that DSS-treated Caco-2 cells disrupt cell homeostasis, accompanied by membrane and protein trafficking impairment, which leads to membrane integrity, cell polarity alteration, and barrier dysfunction [13]. Several studies have reported that honey can improve DSS-induced UC by significantly reducing the levels of the pro-inflammatory factors *IL-6*, *TNF-α*, and *TGF-β1* while upregulating the expression level of *IkB-α* [14]. Additionally, research has shown that honey can alleviate UC by enhancing the antioxidant capacity of cells, including increasing the levels of SOD and GSH-Px [15]. This improvement helps resist intestinal oxidative stress and removes reactive oxygen species (ROS) that accumulate due to disruption of the intestinal epithelial barrier [15].

Ferroptosis is a unique form of cell death linked to oxidative stress and dysregulated iron metabolism [16]. The primary mechanism of ferroptosis involves an imbalance between the generation and degradation of lipid ROS in cells [17]. In this process, the inhibition of glutathione peroxidase (GPX4) reduces the antioxidant capacity of cells, leading to ROS accumulation and oxidative cell death [18]. During inflammation, cells secrete inflammatory mediators, such as *TNF-α*, which contribute to ROS formation, phospholipid peroxidation, and the continuous downregulation of GPX4, thereby accelerating ferroptosis [18]. Recent studies have shown that ferroptosis can alleviate DSS-induced colitis by interfering with the *Nrf2*/*HO-1* signaling pathway [15]. Additionally, it can inhibit endoplasmic reticulum stress through the NF-κB pathway, significantly reducing ferroptosis and alleviating colitis [19]. These findings suggest that blocking the pathogenic pathway of ferroptosis can lead to UC remission [19]. Honey contains functional active substances, such as flavonoids, polyphenols, and amino acids, which exhibit strong antioxidant and anti-inflammatory effects [2]. Studies have found that kaempferol can inhibit ferroptosis by activating the *Nrf2*/*SLC7A11*/GPX4 signaling pathway, while galanin can do so by activating the *SLC7A11*/GPX4 axis [20,21]. Baicalin and safflomin A have been shown to inhibit ferroptosis by upregulating GPX4, inhibiting lipid peroxidation, and reducing iron accumulation [22]. Given the abundance of these functional substances in honey, which can significantly inhibit ferroptosis, it is essential to further explore honey as a natural product with potential therapeutic value for combating ferroptosis.

In this study, we used a DSS-induced Caco-2 cell model to investigate the protective effects of *L. bicolor* honey against UC. Specifically, we focused on how *L. bicolor* honey activates the *Nrf2*/*HO-1* signaling pathway to inhibit ferroptosis. Our evaluation confirmed the anti-inflammatory properties of *L. bicolor* honey in the context of UC. This research provides foundational scientific data on the medicinal value of *L. bicolor* honey and offers a new perspective for UC treatment.

## 2. Methods and Materials

### 2.1. Chemicals and Reagents

The SPE-C_18_ cartridge (Bond Elut-PPL, 500 mg, 6 mL) was purchased from Agilent Technology Co., Ltd. (Santa Clara, CA, USA). Trifolin (purity ≥ 98%) was acquired from Yuanye Biological Technology Co., Ltd. (Shanghai, China). Ferrostatin-1 (Fer-1, purity ≥ 99%) was obtained from Macklin Biochemical Co., Ltd. (Shanghai, China). Dulbecco’s modified Eagle’s medium (DMEM), fetal bovine serum (FBS), 100 U/mL penicillin, and 100 μg/mL streptomycin were sourced from Gibco Life Technologies (New York, NY, USA). Dextran sulfate sodium (DSS, 36,000–50,000 M. W.) was purchased from MP Biomedicals Inc. (Santa Ana, CA, USA). TRIzol reagent, PrimeScript RT Reagent Kit, and SYBR Green Master Mix were obtained from TaKaRa (Dalian, China). NP-40 Buffer was sourced from Solarbio Science & Technology Co., Ltd. (Beijing, China). 2′,7′-dichlorofluorescein diacetate (DCFH-DA) was acquired from Yuanye Biological Technology Co., Ltd. (Shanghai, China). Chromatographic grade methanol, acetonitrile, ethanol, and formic acid were purchased from Thermo Fisher Scientific (Pittsburgh, PA, USA). Ultrapure water was obtained from a Milli-Q Plus System (Burlington, MA, USA).

### 2.2. Honey Sample Preparation

*Lespedeza bicolor* Turcz. honey samples were collected from the northern margin and southeastern hilly area of the Great Khingan Mountains (Heilongjiang, China) from 1 August to 31 August 2020. Based on our previous research, we have confirmed *L. bicolor* honey samples as monofloral honey [23]. The extraction procedure for *L. bicolor* honey was modified based on a previously published method [23]. We started by fully diluting a 5 g *L. bicolor* honey sample with 10 mL of ultrapure water and vortexed for 10 min. Subsequently, the sample underwent centrifugation at 8000× *g* for 5 min to collect the supernatant. Polyphenol compounds in the honey were then collected using SPE-C_18_ cartridges, followed by elution using MeOH. The eluted solution was dried using nitrogen gas. Finally, the dried extracts were dissolved in ultrapure water to obtain a stock solution (at 200 mg/mL) for further study.

### 2.3. Cell Culture Experiments

#### 2.3.1. Cell Culture and Cell Viability Assay

Caco-2 cells were obtained from the Cell Bank of the Shanghai Institute of Biochemistry and Cell Biology, Chinese Academy of Sciences (Shanghai, China). The cells were incubated in DMEM supplemented with 10% FBS (*v*/*v*), 100 U/mL penicillin, and 100 μg/mL streptomycin and maintained in a 5% CO_2_ incubator at 37 °C. Cell viability was assessed using the cell counting kit-8 (CCK-8) from Dojindo, Inc. (Kumamoto, Japan), following the manufacturer’s instructions.

#### 2.3.2. Total RNA Isolation and Quantitation

Caco-2 cells were seeded in 6-well plates at a density of 1 × 10^5^ cells/mL. When cell confluence reached ≥70%, the cells were pretreated with Fer-1 (10 μM), Trifolin (10 μM), and various concentrations of *L. bicolor* honey extract (50, 100, and 200 μg/kg). After 2 h, the cells were stimulated with 2.5% DSS (wt%) for 24 h. The cell medium was discarded, and the cells were washed twice with PBS. Total RNA was extracted from the cells using an RNA extraction system with TRIzol reagent. RNA concentration was measured using a NanoDrop 2000 ultra-micro spectrophotometer from Thermo Fisher Scientific (Pittsburgh, PA, USA). cDNA was synthesized from 1000 ng of total RNA in a 10 μL reaction volume using the PrimeScript RT Reagent Kit (Dalian, China). Quantitative real-time PCR was performed using a 7500c Real-Time PCR Detection System (Hangzhou, China). Gene primers used to amplify selected cytokine transcripts are listed in Table S1. β-actin served as the housekeeping gene for normalization of expression levels. The reaction efficiency of RT-qPCR was shown in Figure S1. The 2^−∆∆Ct^ method was employed to calculate the relative expression levels of the target genes.

#### 2.3.3. Collection of Metabolites from Cells

Caco-2 cells were seeded and pretreated as described in Section 2.3.2. The cell medium was discarded, and cells were washed twice with PBS. Subsequently, 1 mL of a mixed solution of methanol, acetonitrile, and ultrapure water (2:2:1, *v*/*v/v*) was added to the cells, vortexed for 1 min, and centrifuged at 10,000× *g* for 5 min at 4 °C. The supernatant was transferred to a new tube and dried using nitrogen gas. Finally, the cellular extracts were re-dissolved in 200 μL of a mixed solution of acetonitrile and ultrapure water (1:1, *v*/*v*) for UHPLC/Q-TOF-MS analysis.

#### 2.3.4. UHPLC/Q-TOF-MS Analysis

The metabolites of Caco-2 cells were analyzed using an Agilent 1290 Infinity II UHPLC system (Santa Clara, CA, USA) coupled with an Agilent 6545 ESI-Q-TOF high-resolution mass spectrometer (Santa Clara, CA, USA). Chromatographic separation utilized an Agilent ZORBAX Eclipse Plus C_18_ column (2.1 mm × 100 mm, 1.8 μm) (Santa Clara, CA, USA). Mobile phases A and B consisted of water and acetonitrile containing 0.1% formic acid, respectively. The gradient elution program was as follows: 0–2 min, 5% B; 2–20 min, 5%–100% B; 20–25 min, 100% B; followed by a post time of 5 min. The injection volume was 5 μL, and the flow rate was 0.3 mL/min.

For mass spectrometry, the parameters in the negative ionization mode were set as follows: gas temperature, 325 °C; gas flow, 10 L/min; nebulizer pressure, 35 psi; sheath gas temperature, 370 °C; sheath gas flow, 12 L/min; VCap, 3500 V; fragmentor voltage, 135 V. The acquisition range was *m*/*z* 100–1700. The reference ions were set at 112.985587 and 1033.988109 in the negative mode.

#### 2.3.5. Protein Extraction and Analysis of Oxidative Stress Indexes

Caco-2 cells were seeded and pretreated as described in Section 2.3.2. Cells were washed twice with PBS. Caco-2 cells were lysed using 80 μL of NP40 Buffer with a phosphatase inhibitor cocktail. The protein concentration was determined using the BCA Protein Assay Kit from Beyotime Biotechnology Co., Ltd. (Shanghai, China), according to the manufacturer’s instructions.

Oxidative stress markers included superoxide dismutase (SOD) activity, malondialdehyde (MDA) content, total iron content, and glutathione (GSH) content. Kits used for analysis included the Total Superoxide Dismutase Assay Kit with WST-8, Lipid Peroxidation Assay Kit, and Cellular Glutathione Peroxidase Assay Kit with DTNB, all from Beyotime Biotechnology Co., Ltd. (Shanghai, China). The total iron content in the cells was determined using an iron assay kit from Yuanye Biological Technology Co., Ltd. (Shanghai, China). These kits were used to measure SOD activity, MDA content, iron ion content, and GSH content in Caco-2 cells, according to the manufacturer’s instructions.

#### 2.3.6. Detection of Cellular ROS Generation

Caco-2 cells were seeded and pretreated as described in Section 2.3.2. The cell slides were washed with PBS and then incubated with 10 μM DCFH-DA for 30 min at 37 °C. Subsequently, the cells were washed twice with PBS and examined using a confocal laser scanning microscope from Leica Camera AG (Hessian, Germany) with excitation/emission at 480/520 nm.

### 2.4. Statistical Analysis

Variance testing was performed using IBM SPSS version 26.0 software (New York, NY, USA). Data were presented using GraphPad Prism 8.0 (San Diego, CA, USA). Agilent MassHunter Profinder B.10.0 software from Agilent Technologies Co., Ltd. (Santa Clara, CA, USA) was used to convert the data acquisition.d files into .cef files. Data analysis was conducted using Agilent Mass Profiler Professional 15.0 software (MPP) from Agilent Technologies Co., Ltd. (Santa Clara, CA, USA), with the parameters set according to previous research [23].

Following this, partial least squares discriminant analysis (PLS-DA) and volcano plots were used to identify distinct features among different treatments, performed using SIMCA software version 14.1 from Sartorius Stedim (Göttingen, Germany). Metabolic pathway analyses were conducted using MetaboAnalyst 4.0 (https://www.metaboanalyst.ca, accessed on 2 December 2023) and KEGG (http://www.kegg.jp). All experiments were performed in triplicate.

## 3. Results and Discussion

### 3.1. Effect of Different Treatments on Caco-2 Viability

**Figure 1:**
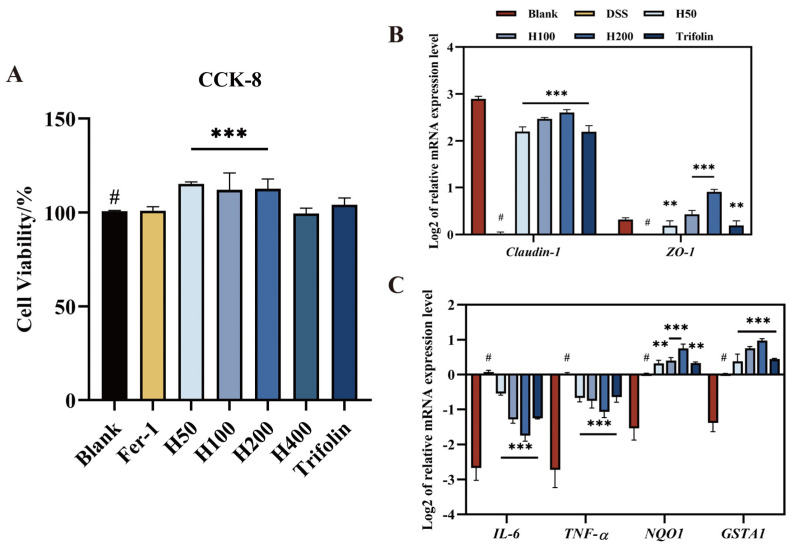
Effects of different treatments on Caco-2 cell viability, tight junction cytokines, and inflammation factors in DSS-induced Caco-2 cells. (**A**) Effect of the *L. bicolor* honey extract (50, 100, 200, and 400 μg/kg), Fer-1 (10 μM), and Trifolin (10 μM) on the cell viability of Caco-2 cells. (**B**) Effect of the *L. bicolor* honey extract (50, 100, and 200 μg/kg) and Trifolin (10 μM) on the mRNA expression of the tight junction genes *Claudin-1* and *ZO-1* in DSS-induced Caco-2 cells. (**C**) Effect of the *L. bicolor* honey extract (50, 100, and 200 μg/kg) and Trifolin (10 μM) on the mRNA expression of the inflammation genes *IL-6*, *TNF-α*, *NQO1*, and *GSTA1* in DSS-induced Caco-2 cells. ** *p* < 0.01, *** *p* < 0.001 compared with ‘#’, representing the Blank group.

To ensure that all treatments did not cause obvious toxic effects on Caco-2 cell growth and metabolism, we used the CCK-8 assay to assess the impact of different concentrations of *L. bicolor* honey extract (50, 100, 200, and 400 μg/kg), Fer-1 (10 μM), Trifolin (10 μM), and without any treatment (the Blank group) on Caco-2 cell viability. The results are shown in Figure 1A. Compared to the Blank group, pretreatment with *L. bicolor* honey extract at 50, 100, and 200 μg/kg significantly increased cell viability (*p* < 0.001). However, at a concentration of 400 μg/kg, *L. bicolor* honey extract did not significantly affect cell viability. The treatment groups with 10 μM Fer-1 and 10 μM Trifolin did not significantly alter cell viability. Therefore, we selected *L. bicolor* honey extract (50, 100, and 200 μg/kg), Fer-1 (10 μM), and Trifolin (10 μM) for subsequent experiments.

### 3.2. Effect of Different Treatments on Expression of Relative Tight Junction Cytokines

DSS-induced UC stimulates the colonic mucosa and increases intestinal barrier permeability, resulting in downregulation of tight junction proteins that act as intestinal barriers, such as *ZO-1*, *ZO-2*, *Occludins*, and *Claudins* [24]. We established a model of DSS-induced intestinal barrier dysfunction in Caco-2 cells. *L. bicolor* honey extract, which contains Trifolin as its primary biomarker [23], was used to pretreat Caco-2 cells to assess its effect.

The mRNA transcription levels of tight junction cytokines *ZO-1* and *Claudin-1* were measured by qPCR to investigate the protective effect of *L. bicolor* honey on intestinal barrier integrity. The results are shown in Figure 1B. Initially, cells were pretreated with 2.5% DSS, which disrupted the integrity of the monolayer and significantly downregulated *ZO-1* and *Claudin-1* expression (*p* < 0.001). In the Blank group, their expression levels were significantly higher than those in the DSS-treated group (*p* < 0.001). Subsequently, Caco-2 cells were incubated with different concentrations of *L. bicolor* honey extract (50, 100, and 200 μg/kg) and 10 μM Trifolin. Compared to the DSS group, the expression of the tight junction cytokine was significantly upregulated in the treatment groups (*p* < 0.001), with a concentration-dependent effect observed for *L. bicolor* honey. A decrease in the expression levels of *Claudin-1* and *ZO-1* can lead to the impairment of tight junction function, improve the permeability of intestinal tissue, and eventually lead to the occurrence of various systemic infectious diseases and even tumors. The increase in the expression levels of *Claudin-1* and *ZO-1* means a significant inhibitory effect on the development of inflammatory diseases. Additionally, the effect of 10 μM Trifolin was not significantly different from that of 100 μg/kg *L. bicolor* honey extract, suggesting similar efficacy between the two treatments. In conclusion, *L. bicolor* honey extract and Trifolin have a significant protective effect on the tight junctions of Caco-2 cells.

### 3.3. Effect of Different Treatments on Inflammation Cytokines Expression

UC pathogenesis stimulates an inflammatory response, causing damage to the intestinal mucosa, compromising the intestinal mucosal barrier, and facilitating the invasion of external toxins and microorganisms, resulting in the release of endotoxins. This process stimulates the expression of inflammatory factors, such as *TNF* and interleukin (*IL*); compared with the Blank group, its expression was significantly increased (*p* < 0.001), thereby exacerbating intestinal damage [25]. *TNF-α* and *IL-6* are critical transcription factors that regulate these inflammatory responses [14]. *TNF-α*, in particular, is the main pro-inflammatory factor contributing to intestinal barrier dysfunction, while *IL-6* is synthesized under inflammatory conditions and exacerbates intestinal inflammation [14]. In the 2.5% DSS-induced Caco-2 cell monolayer inflammation model, Caco-2 cells were pretreated with different concentrations of *L. bicolor* honey extract (50, 100, and 200 μg/kg) and 10 μM Trifolin. The mRNA transcription levels of the inflammatory mediators *IL-6* and *TNF-α* were assessed using qPCR technology to investigate the inhibitory effect of *L. bicolor* honey on the intestinal inflammatory response. The results are presented in Figure 1C. Initially, 2.5% DSS was used to induce an inflammatory response in the Caco-2 cells.

Compared to the Blank group, the inflammatory mediators *IL-6* and *TNF-α* were significantly upregulated (*p* < 0.001). Subsequently, Caco-2 cells were treated with different concentrations of *L. bicolor* honey extract (50, 100, and 200 μg/kg) and 10 μM Trifolin. The expression of both inflammatory mediators was significantly downregulated compared to that in the DSS group (*p* < 0.001), with the effect showing a concentration-dependent response to *L. bicolor* honey. Additionally, the effect of 10 μM Trifolin and 100 μg/kg *L. bicolor* honey extract on *IL-6* showed no significant difference, and similarly, the effects of 10 μM Trifolin and 50 μg/kg *L. bicolor* honey extract on *TNF-α* were not significantly different. These results indicate that Trifolin and *L. bicolor* honey extract have similar effects in reducing inflammatory factors. *TNF-α* and *IL-6* are important cytokines involved in the maintenance and dynamic balance of the immune system, and they can regulate inflammation and host defense. The expression of *TNF-α* and *IL-6* was disordered under the stimulation of DSS but tended to be normal after treatment with *L. bicolor* honey and Trifolin. In conclusion, *L. bicolor* honey extract and Trifolin significantly downregulate the expression levels of the inflammatory mediators *IL-6* and *TNF-α* during the inflammatory response, thereby regulating the inflammatory process.

### 3.4. Effect of Different Treatments on Antioxidant Factors Expression

The pathogenesis of UC is closely linked to the oxidative stress response [26]. Upon activation by oxidative stress, cells produce numerous reactive oxygen species and electrophiles, disrupting the original redox balance [27]. *Quinone oxidoreductase 1* (*NQO1*) plays a crucial role in reducing oxidative stress by regulating reduced coenzyme Q and vitamin E [28]. *GSTA1*, a member of the glutathione transferase (GST) family, is one of the most abundant detoxification enzymes. Its expression enhances cellular fusion, influencing the cell’s response to oxidative stress [29].

To establish a monolayer oxidative stress model, Caco-2 cells were induced with 2.5% DSS. Different concentrations of *L. bicolor* honey extract (50, 100, and 200 μg/kg) and 10 μM Trifolin were used for pretreatment. The mRNA expression levels of the antioxidant factors *NQO1* and *GSTA1* were assessed using qPCR technology. The results are shown in Figure 1C. The expression levels of *NQO1* and *GSTA1* in Caco-2 cells induced by 2.5% DSS were significantly upregulated compared to those in the Blank group (*p* < 0.001). Furthermore, compared to the DSS group, the expression of *NQO1* and *GSTA1* was significantly upregulated in Caco-2 cells treated with different concentrations of *L. bicolor* honey extract and Trifolin (*p* < 0.01), demonstrating a concentration-dependent effect of *L. bicolor* honey. Cells under the effect of DSS stimulate the oxidative stress reaction. GSTA1 can convert and metabolize reactive oxygen species (ROS) to control cell damage by free radicals and peroxides in the oxidative stress response. *GSTA1* and *NQO1* are antioxidant factors that maintain redox homeostasis. The antioxidant effect of 10 μM Trifolin and 100 μg/kg *L. bicolor* honey extract on *NQO1* and *GSTA1* showed no significant difference, indicating a similar antioxidant effect between the two treatments. In conclusion, *L. bicolor* honey extract and Trifolin play a regulatory role in the antioxidant response to DSS-induced oxidative stress and maintain cellular redox homeostasis.

### 3.5. Effect of Different Treatments on Cell Metabolism

**Figure 2:**
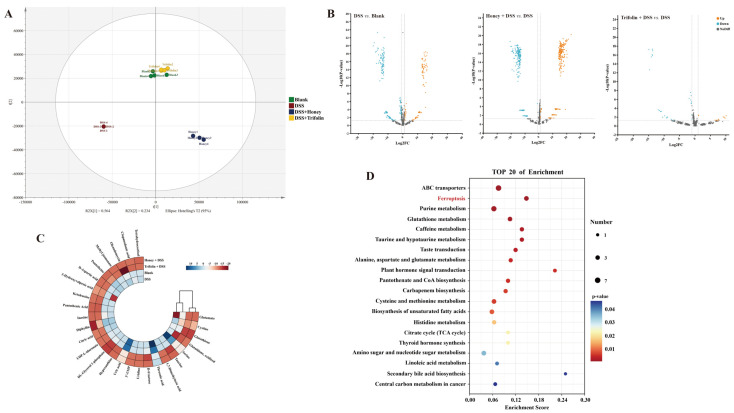
Effects of different treatments on cellular metabolites in DSS-induced Caco-2 cells. (**A**) Partial least squares discrimination analysis (PLS-DA) based on the metabolites of Caco-2 cells among Blank, DSS, 100 μg/kg Honey + DSS, and 10 μM Trifolin + DSS groups (n = 4 for each group). (**B**) Volcano plot based on the differential metabolites of Caco-2 cells among DSS vs. Blank, 100 μg/kg Honey + DSS vs. DSS, and 10 μM Trifolin + DSS vs. DSS. (**C**) Log_2_ value of differential metabolites based on *p* value < 0.05, VIP ≥ 1 from PLS-DA, and FoldChange ≥ 2 or ≤0.5 from the volcano plot among each group. (**D**) Top-20 metabolic pathway mappings with KEGG databases based on FoldChange ≥ 2 or ≤0.5 for the difference in metabolites among each group.

The PLS-DA model in Figure 2A demonstrated significant changes in Caco-2 cells after 2.5% DSS induction compared to the Blank group (*p* < 0.001). Compared to the DSS group, cell metabolites were significantly altered following treatment with 100 μg/kg *L. bicolor* honey extract (*p* < 0.001) and 10 μM Trifolin (*p* < 0.001). This indicates that *L. bicolor* honey extract and Trifolin can mitigate the effects of UC induced by DSS. Subsequently, we utilized a volcano plot to compare differential metabolites between *L. bicolor* honey extract and Trifolin-treated groups with the DSS group, as well as between the Blank group and DSS group, as shown in Figure 2B. Differential metabolites were filtered based on FoldChange ≥ 2 or FoldChange ≤ 0.5 and differential metabolites with VIP ≥ 1, as determined by the PLS-DA model [30]. Additionally, differential metabolites were further filtered based on *p* < 0.05 (ANOVA) to identify compounds, as shown in Table 1. The distribution of content in each group of these differential metabolites is depicted in Figure 2C.

The differential metabolites were mapped to the KEGG Pathway database to elucidate their involvement in cellular pathways. The top 20 enriched pathways are depicted in Figure 2D. The significance of these enriched pathways was determined by calculating their *p* value and RichFactor using the KEGG Pathway database. A smaller *p* value and a higher RichFactor indicate a greater degree of influence of the pathway [31]. Additionally, the numerical value assigned to each pathway indicates the number of compounds affected, with a higher number reflecting a more substantial impact on the pathway [31]. Through this analysis, pathways highly influential and associated with intestinal inflammation were scrutinized, ultimately identifying ferroptosis as a significant pathway [15].

Table 1 presents the differential metabolites observed in Caco-2 cells induced by DSS, in which pretreatment with 100 μg/kg *L. bicolor* honey extract and 10 μM Trifolin primarily influenced the levels of glutamate, cystine, GSH, and GSSG. Compared to the Blank group, the expression of cystine was downregulated, while the expression of other substances was upregulated in the DSS group. Following pretreatment with *L. bicolor* honey extract and Trifolin, the expression of these substances was downregulated compared to that in the DSS group. These findings suggest that both *L. bicolor* honey extract and Trifolin attenuate the ferroptosis process in DSS-induced colitis.

Amino acid metabolism plays a pivotal role in the intricate pathways of ferroptosis [32]. Oxidative stress induced by excess glutamate stands as a key initiator in the activation of ferroptosis pathways [33]. Fueled by heightened concentrations of glutamate within cells, System Xc, situated on the cell membrane, orchestrates the transport of extracellular cystine and intracellular glutamate [34]. When cells encounter oxidative stress, the extracellular glutamate levels surge, thereby hindering the transport functionality of System Xc [34]. As shown in Table 1 and Figure 2C, following DSS induction in Caco-2 cells, there was a downregulation in cystine expression and an upregulation in glutamate expression. These findings underscore that escalated extracellular glutamate under oxidative stress curtails the transport capabilities of System Xc, consequently diminishing cystine uptake rates and impeding glutamate exportation from cells, leading to intracellular accumulation.

Table 1 exhibits the downregulation of intracellular and extracellular glutamate expression subsequent to treatment with *L. bicolor* honey extract and Trifolin. This observation suggests mitigation of the oxidative stress response in Caco-2 cells, resulting in reduced extracellular glutamate levels and restoration of System Xc transport functionality, thereby decreasing intracellular glutamate levels. However, compared to the DSS group, the downregulation of intracellular cystine expression suggests that *L. bicolor* honey extract and Trifolin may not fully restore Caco-2 cells to their normal state.

### 3.6. Effect of Different Treatments on Oxidative Stress

**Figure 3:**
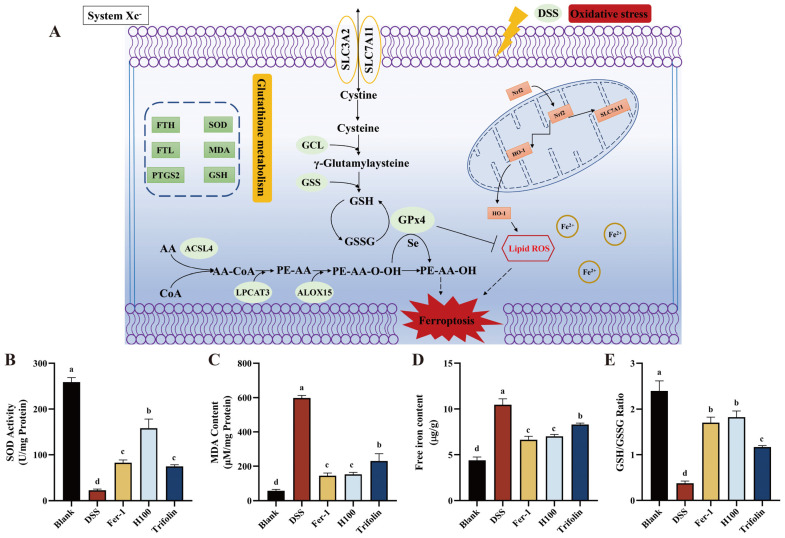
(**A**) Schematic diagram of the mechanism of ferroptosis in DSS-induced human Caco-2 cells through the *Nrf2*/*HO-1* pathway. Effects of different treatments on oxidative stress in DSS-induced Caco-2 cells. Content of (**B**) SOD, (**C**) MDA, (**D**) free iron, and (**E**) GSH/GSSG ratio in DSS-induced Caco-2 cells. a, b, c, and d indicate significant differences (*p* < 0.05). The groups containing differently labeled letters indicate significant differences (*p* < 0.05). The groups with the same letter labeled exhibit no significant differences.

Previous studies have demonstrated that MDA, GSH, and iron ion loading serve as three markers of ferroptosis [35]. A key characteristic of ferroptosis is lipid peroxidation, resulting in the cytotoxic byproduct MDA [35]. As illustrated in Figure 3B, compared to the Blank group, the MDA content in Caco-2 cells significantly increased following induction with 2.5% DSS (*p* < 0.001). Pretreatment of Caco-2 cells with 100 μg/kg *L. bicolor* honey extract and 10 μM Trifolin markedly decreased MDA content compared to the DSS group (*p* < 0.001). Moreover, there was no significant difference in MDA inhibition between the *L. bicolor* honey extract and Fer-1 groups. These findings suggest that *L. bicolor* honey extract and Trifolin can alleviate DSS-induced colitis by reducing MDA content and inhibiting lipid peroxidation induced by inflammation.

Excessive accumulation of peroxide in cells is directly associated with ferroptosis [36]. As shown in Table 1, the expression of GSH and GSSG was upregulated following DSS induction, which is attributable to the oxidative stress response that generates peroxide in cells [37]. Consequently, GSH levels were elevated, providing hydrogen electrons and participating in redox reactions, thereby leading to increased GSSG production. Pretreatment of Caco-2 cells with *L. bicolor* honey extract inhibited the inflammatory response, resulting in the downregulation of GSH and GSSG expression. These findings indicate that *L. bicolor* honey possesses antioxidant capacity, promoting peroxidase removal from cells and adjusting GSH biosynthesis and metabolic disorders to resist DSS-induced oxidative stress.

Additionally, we evaluated the GSH/GSSG ratio, as shown in Figure 3E. After DSS-induced Caco-2 cells, compared to the Blank group, higher levels of oxidative stress were observed, leading to a decreased GSH/GSSG ratio (*p* < 0.001). Pretreatment with *L. bicolor* honey extract and Trifolin significantly increased the GSH/GSSG ratio compared to the Blank group (*p* < 0.001). These results further validate the findings of Section 3.5, demonstrating that *L. bicolor* honey extract and Trifolin have a detoxifying effect on DSS-induced Caco-2 cells and enhance cellular redox levels. It has been shown that *L. bicolor* honey extract and Trifolin protect GSH against oxidative and free radical-mediated cell damage.

Iron ions play a critical role in ferroptosis and are essential components of lipid peroxidases, catalyzing redox reactions in cells [38]. Fe^2+^, due to its instability and high reactivity, can generate hydroxyl radicals via the Fenton reaction and directly react with polyunsaturated fatty acids in membranes, thereby producing a large number of ROS that lead to ferroptosis [38,39]. Following induction with 2.5% DSS, the iron ion content in Caco-2 cells was significantly higher compared to that in the Blank group (*p* < 0.001). However, pretreatment with 100 μg/kg *L. bicolor* honey extract and 10 μM Trifolin significantly decreased the iron ion content compared to the DSS group (*p* < 0.001). Furthermore, there was no significant difference between the *L. bicolor* honey extract treatment group and the Fer-1 treatment group. These findings suggested that *L. bicolor* honey extract and Trifolin protect against DSS-induced colitis by reducing intracellular iron ion levels and inhibiting ferroptosis.

SOD is a crucial antioxidant metalloenzyme in the body, playing a pivotal role in maintaining the balance between oxidation and antioxidation [40]. Previous studies have demonstrated its significant role in colitis by scavenging superoxide free radicals, thereby inhibiting inflammation and apoptosis and ameliorating colitis [41]. A colitis model was established in Caco-2 cells by stimulation with 2.5% DSS. Compared to the Blank group, the SOD content in the DSS group was significantly decreased (*p* < 0.001). Pretreatment of Caco-2 cells with 100 μg/kg *L. bicolor* honey extract and 10 μM Trifolin resulted in a substantial increase in SOD content compared to the Blank group. Moreover, *L. bicolor* honey extract significantly increased the SOD content compared to that in the Fer-1 group (*p* < 0.05). These findings indicate that *L. bicolor* honey extract can modulate SOD content, restore cellular redox balance, and mitigate the ferroptosis process during DSS-induced cellular inflammation.

**Figure 4:**
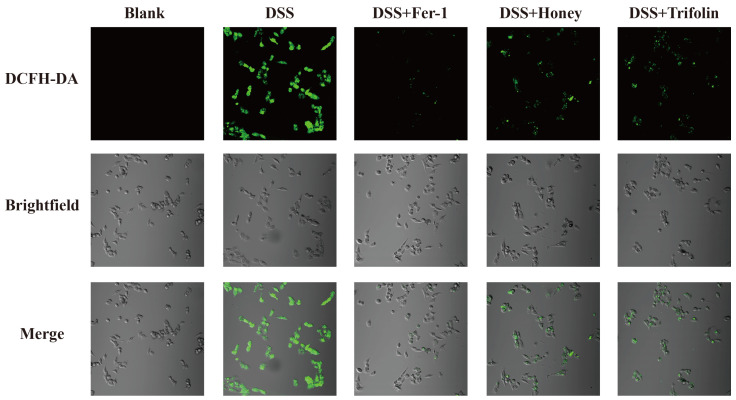
Effects of different treatments on the ROS expression level in DSS-induced Caco-2 cells. ‘DCFH-DA’ indicated that ROS was captured via a confocal laser scanning microscope at a 488 nm excitation wavelength and a 525 nm emission wavelength. The scale bar is 5 μm and the magnification is 200×. ‘Brightfield’ indicated that images were captured by a normal light source. ‘Merge’ indicated the superposition of two images, ‘DCFH-DS’ and ‘Brightfield’.

One characteristic of ferroptosis is the imbalance between the generation and degradation of lipid ROS in cells [38]. This imbalance is primarily caused by the excessive accumulation of intracellular iron-dependent ROS and the reduced scavenging effect of GPX4 [42]. When the antioxidant capacity of cells decreases and becomes insufficient to remove the excessive accumulation of lipid ROS, ferroptosis occurs [38]. To evaluate this, we measured intracellular ROS levels. As depicted in Figure 4, fluorescence intensity was significantly higher in the DSS group compared to the Blank group, indicating activation of ferroptosis in Caco-2 cells under DSS induction. However, fluorescence intensity markedly decreased following pretreatment with 10 μM Fer-1, 100 μg/kg *L. bicolor* honey extract, and 10 μM Trifolin. This suggests that these treatments can inhibit lipid oxidation and reduce ROS levels, thereby regulating ferroptosis and mitigating the inflammatory response induced by DSS.

### 3.7. Effect of Different Treatments on Ferroptosis through Nrf2/HO-1 Pathway

*The Nrf2*/*HO-1* pathway is a critical signaling pathway involved in the antioxidative stress response, and its upregulation can protect intestinal cells [43]. When DSS-induced oxidative stress occurs in monolayer enterocytes, *Nrf2* dissociates from KEAP1 and translocates to the nucleus, where it becomes activated [44]. Once in the nucleus, *Nrf2* binds to ARE and promotes the expression of downstream genes and antioxidant enzymes, such as *NQO1* and *HO-1*, thereby regulating cellular homeostasis [45].

**Figure 5:**
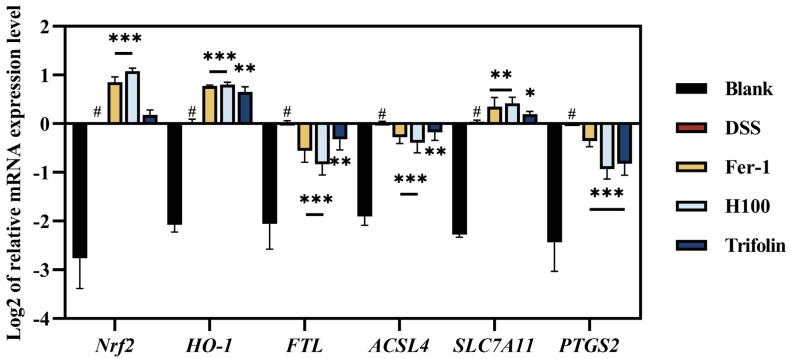
*L. bicolor* honey and Trifolin prevents ferroptosis by *Nrf2*/*HO-1* pathway in DSS-induced Caco-2 cells. Effect of *L. bicolor* honey extract (100 μg/kg), Fer-1 (10 μM), and Trifolin (10 μM) on mRNA expression of the relative genes for *Nrf2*/*HO-1* and the ferroptosis pathway, including *Nrf2*, *HO-1*, *FTL*, *ACSL4*, *SLC7A11*, and *PTGS2* in DSS-induced Caco-2 cells. * *p* < 0.05, ** *p* < 0.01, *** *p* < 0.001 compared with ‘#’, representing the Blank group.

As depicted in Figure 5, the induction of Caco-2 cells with 2.5% DSS significantly increased the expression of *Nrf2* compared to the Blank group (*p* < 0.001). Treatment with 10 μM Fer-1, 100 μg/kg *L. bicolor* honey extract, and 10 μM Trifolin further increased *Nrf2* expression compared to that in the DSS group. Moreover, there was no significant difference in *Nrf2* expression between the groups treated with 10 μM Fer-1 and 100 μg/kg *L. bicolor* honey extract. *HO-1* is an important antioxidant enzyme that degrades heme, plays an antioxidant role, and breaks down biliverdin and its reduced products, such as bilirubin and iron ions [46]. 

HO-1 can remove ROS and participate in the process of ferroptosis through its antioxidant action [47]. As illustrated in Figure 5, following stimulation with 2.5% DSS, the expression of *HO-1* was significantly increased compared to that in the Blank group (*p* < 0.001). Subsequent pretreatment of cells with 10 μM Fer-1, 100 μg/kg *L. bicolor* honey extract, and 10 μM Trifolin further significantly increased *HO-1* expression compared to the DSS group (*p* < 0.01). Moreover, there was no significant difference in *HO-1* expression between the groups treated with 10 μM Fer-1 and 100 μg/kg *L. bicolor* honey extract. The expression level of *Nrf2* can affect the expression of *HO-1*. In addition, *HO-1,* the product of the degradation of bilirubin nitric oxide and other *Nrf2* regulation of genes for UC, has a regulatory role. These findings suggested that *L. bicolor* honey extract and Trifolin could regulate the ferroptosis process and protect against DSS-induced colitis by upregulating the *Nrf2*/*HO-1* pathway to exert antioxidant effects.

### 3.8. Effect of Different Treatments on Intestinal Epithelial Cell Ferroptosis

Furthermore, we detected the expression levels of several key factors (*FTL*, *ACSL4*, *SLC7A11*, and *PTGS2*) in ferroptosis by qPCR technology to validate the above results and evaluate the regulatory effects of *L. bicolor* honey extract and Trifolin on the ferroptosis process.

For *FTL*, as one of the subunits of ferritin, excess intracellular iron ions can be stored in ferritin, isolating them and preventing them from participating in ROS generation reactions [48]. When disruption of ferritin leads to elevated ROS and activation of ferroptosis, *FTL* bound to iron ions dissociates and its content increases [48]. As shown in Figure 5, following induction with 2.5% DSS, the expression level of *FTL* in Caco-2 cells significantly increased compared to that in the Blank group (*p* < 0.001). However, its expression level decreased in the treatment group compared to that in the DSS group (*p* < 0.01).

For *ACSL4*: Its mechanism involves promoting the esterification of polyunsaturated fatty acids (PUFA) to acyl-coenzyme A (acyl-CoA), thereby promoting ferroptosis [49]. As depicted in Figure 5, following induction with 2.5% DSS, the expression of *ACSL4* in Caco-2 cells significantly increased compared to that in the Blank group (*p* < 0.001), indicating induction of ferroptosis. Compared to the DSS group, the expression level of *ACSL4* was decreased in the treatment group (*p* < 0.01). Therefore, as an important target for treating ferroptosis-related diseases, the expression level of *ACSL4* can be reduced by the intervention of *L. bicolor* honey and Trifolin to slow down the progression of ferroptosis-related diseases.

For *SLC7A11*, as the transport functional unit of the Xc^−^ system, it plays a crucial role in the reverse transport of cystine and glutamate across the cell membrane, facilitating the uptake of cystine and its conversion into cysteine inside the cell, a key step in GSH synthesis [50]. GSH, in turn, participates in reducing lipid peroxides to mitigate ROS production, thereby preventing ferroptosis [37].

As depicted in Figure 5, following stimulation of Caco-2 cells with 2.5% DSS, the expression of *SLC7A11* was significantly decreased (*p* < 0.001). This result indicated that DSS induction led to reduced *SLC7A11* expression, impaired Xc^−^ system function, and disruption of lipid redox balance, ultimately leading to ferroptosis [50]. Following intervention with different treatment groups, the expression levels of *SLC7A11* were significantly higher compared to those in the DSS group (*p* < 0.001).

These results indicated that the Xc^−^ system restored its function, intracellular lipid redox returned to normal, and ferroptosis was inhibited. *PTGS2* is considered one of the markers of ferroptosis and is involved in the inflammatory response, enhancing the release of *TNF-α*, *IL-1β*, *IL-6*, and other inflammatory factors and promoting ferroptosis [51]. As shown in Figure 5, Caco-2 cells stimulated with 2.5% DSS showed significantly decreased expression (*p* < 0.001). After intervention with different treatment groups, its expression levels were significantly lower than those in the DSS group (*p* < 0.001). These results, consistent with Figure 1C, indicated that *L. bicolor* honey extract and Trifolin inhibited ferroptosis. Additionally, after intervention with different treatment groups, the expression of the inflammatory factors *TNF-α* and *IL-6* in Caco-2 cells significantly decreased (*p* < 0.01), as shown in Figure 1C. Therefore, *L. bicolor* honey extract and Trifolin could alleviate DSS-induced colitis by inhibiting the release of inflammatory factors and thereby inhibiting ferroptosis.

## 4. Conclusions

This study further explores the anti-inflammatory effects and regulatory mechanisms of *L. bicolor* honey and its biomarker, Trifolin. Using a DSS-induced Caco-2 model, we found that pretreatment with *L. bicolor* honey extract and Trifolin increased the tight junction integrity of damaged cells and maintained the integrity of the monolayer. Additionally, *L. bicolor* honey extract and Trifolin demonstrated anti-inflammatory effects by reducing the expression of inflammatory factors *TNF-α* and *IL-6* and antioxidant effects by increasing the expression of antioxidant factors *NQO1* and *GSTA1*. Metabolomic analysis revealed that *L. bicolor* honey extract and Trifolin could regulate ferroptosis by reducing SOD, MDA, iron ion load, and ROS release and by modulating the gene expression levels of ferroptosis-related factors *FTL*, *ACSL4*, *SLC7A11*, and *PTGS2* to alleviate the DSS-induced ferroptosis-related inflammatory response. Furthermore, our results suggest that *L. bicolor* honey extract and Trifolin inhibit ferroptosis by activating the *Nrf2*/*HO-1* pathway. These findings provide foundational scientific data supporting the potential application of *L. bicolor* honey as a natural anti-inflammatory agent for human health.

## Figures and Tables

**Table 1:** Comparison of the variations in cellular metabolites across Caco-2 cells for each of the three designated groups.

Metabolite	Formula	Retention Time	*m*/*z*	VIP	DSS vs. Blank			Honey + DSS vs. Blank			Trifolin vs. Blank		
					Trend	Log2FC	*p* Value	Trend	Log2FC	*p* Value	Trend	Log2FC	*p* Value
Glutamate	C_5_H_9_NO_4_	0.73	146.05	10.0575	up	18.34	0.0000	down	−12.74	0.0170	down	−3.1901	0.0318
Cystine	C_6_H_12_N_2_O_4_S_2_	0.74	239.02	8.5470	down	−1.89	0.0398	down	−6.52	0.0003	down	−6.5229	0.0495
Glutathione	C_13_H_22_N_4_O_8_S_2_	0.75	425.08	7.9146	up	2.11	0.0000	down	−2.11	0.0000	down	−2.1085	0.0341
Glutathione oxidized	C_20_H_32_N_6_O_12_S_2_	0.84	611.15	6.1128	up	0.64	0.0001	down	−15.92	0.0000	down	−15.9153	0.0000
Serine	C_3_H_7_NO_3_	0.71	104.04	5.3716	down	−12.84	0.0000	down	−6.45	0.0062	down	−6.4452	0.0494
Taurine	C_2_H_7_NO_3_S	0.73	124.01	5.0767	down	−0.20	0.0064	down	−0.06	0.0233	down	−0.064	0.0083
1,7-Dimethyluric acid	C_7_H_8_N_4_O_3_	0.74	195.05	5.0691	down	−0.73	0.0311	up	0.21	0.0001	up	0.2116	0.0331
Threonic acid	C_4_H_8_O_5_	0.74	195.05	4.8817	down	−13.74	0.0119	down	−9.12	0.0000	down	−9.1242	0.0102
D-Fructose	C_6_H_12_O_6_	0.79	179.06	4.1736	down	−0.26	0.0341	down	−1.97	0.0000	down	−1.974	0.0412
Uridine	C_9_H_12_N_2_O_6_	0.81	243.06	3.9907	down	−0.26	0.0050	down	−0.11	0.0000	down	−0.1123	0.0063
3′-UMP	C_9_H_13_N_2_O_9_P	0.84	323.03	3.9316	down	−0.91	0.0005	up	0.88	0.0000	up	0.0255	0.0082
Uric acid	C_5_H_4_N_4_O_3_	0.84	167.02	3.4736	down	−10.34	0.0032	down	−9.40	0.0209	down	−9.4025	0.0055
Hypoxanthine	C_5_H_4_N_4_O_3_	0.88	135.03	3.3181	down	−0.30	0.0234	up	0.01	0.0000	up	6.0114	0.0097
DL-Glycerol 1-phosphate	C_3_H_9_O_6_P	0.88	171.01	3.3044	down	−0.51	0.0102	down	−2.00	0.0001	down	−1.9774	0.0417
UDP-L-iduronate	C_15_H_22_N_2_O_18_P	0.90	579.03	3.2945	down	−10.04	0.0004	down	−7.44	0.0030	down	−7.441	0.0494
Citric acid	C_6_H_8_O_7_	0.91	191.02	3.1514	down	−1.68	0.0056	down	−0.65	0.0000	down	−0.6549	0.0127
Diphyllin	C_21_H_16_O_7_	0.91	379.08	3.1118	down	−2.40	0.0197	up	3.04	0.0455	up	3.043	0.0478
Inosine	C_10_H_12_N_4_O_5_	1.11	267.07	3.0353	down	−0.47	0.0068	up	2.90	0.0000	up	2.8998	0.0345
Pantothenic acid	C_9_H_17_NO_5_	1.96	218.10	2.9245	down	−0.14	0.0006	up	0.25	0.0000	up	0.2456	0.0061
Ketoleucine	C_6_H_10_O_3_	4.30	129.06	2.7742	down	−0.07	0.0010	up	2.84	0.0000	up	0.4719	0.0345
3-Hydroxyvalproic acid	C_8_H_16_O_3_	8.17	159.10	2.5278	down	−8.56	0.0003	up	1.81	0.0377	up	10.8114	0.0005
D-Aspartic acid	C_4_H_7_NO_4_	8.97	192.05	2.4500	down	−0.85	0.0341	up	−0.27	0.0000	up	−0.2678	0.0213
Pantetheine	C_11_H_23_N_2_O_7_P	10.23	403.09	2.0918	down	−2.05	0.0069	up	2.13	0.0006	up	0.0000	0.0001
Methyl jasmonate	C_13_H_20_O_3_	11.18	223.14	1.8921	down	−0.02	0.0334	up	0.01	0.0000	up	0.0141	0.0079
Oleandomycin	C_35_H_61_NO_12_	16.17	732.41	1.8276	down	−2.14	0.0000	down	−2.02	0.0000	down	−2.0226	0.0057
Clupanodonic acid	C_22_H_34_O_2_	18.40	329.25	1.7231	up	1.62	0.0104	down	−0.35	0.0000	down	−0.1832	0.0199
Tetrahydrocortisol	C_21_H_33_FO_5_	20.73	443.25	1.6974	down	−0.62	0.0000	up	2.09	0.0000	up	−0.3462	0.0172

## Data Availability

The data presented in this study are available in the article and Supplementary Materials.

## Supplementary Materials

The following supporting information can be downloaded at: https://www.mdpi.com/article/10.3390/antiox13080900/s1. Table S1. The gene primers for targeted cytokines. Figure S1. Standard curves for reaction efficiency of q-PCR.
