# Supplementary material for: Lespedeza bicolor Turcz. Honey Prevents Inflammation Response and Inhibits Ferroptosis by Nrf2/HO-1 Pathway in DSS-Induced Human Caco-2 Cells
Source: Antioxidants (Basel). 2024 Jul 25;13(8):900. doi: 10.3390/antiox13080900 (PMC11351236; doi:10.3390/antiox13080900)
Supplement: Supplementary file 1 [file antioxidants-13-00900-s001.zip › antioxidants-3092749-supplementary.pdf]

Table S1. The gene primers for targeted cytokines.

Gene	Primers (5' to 3')	Primers (3' to 5')
β-actin	agttgcgttacaccctttcttg	tcaccttcaccgttccagttt
Claudin-1	tgttgggcttcattctcg	ccaccatcaaggcacgg
ZO-1	agatgaacgggctacgc	ggagactgccattgcttg
IL-6	ctctgcaagagacttccatcc	gaattgccattgcacaactc
TNF-α	ctccagtggctgaaccgc	ggtaggagacggcgatgc
NQO1	tggtgaacagttttggcataa	tcagcatctggtaaaggagg
GSTA1	atggttgagattgatgggatg	aggcagggaagtagcgatt
FTL	cagcctggtcaatttgtacct	gccaattcgcggaagaagtg
ACSL4	tgggctaaatgaatctgaggcttcc	ggcgttggtctacttggaggaatg
SLC7A11	caaatgcagtggcagtgacctt	accgttcatggagccaaagc
PTGS2	ctggcgctcagccatacag	cgcacttatactggtcaaatccc
Nrf2	ccagcccagcacatccag	cgtagccgaagaaacctcat
HO-1	caggcagagaatgctgag	gcttcacatagcgctgca

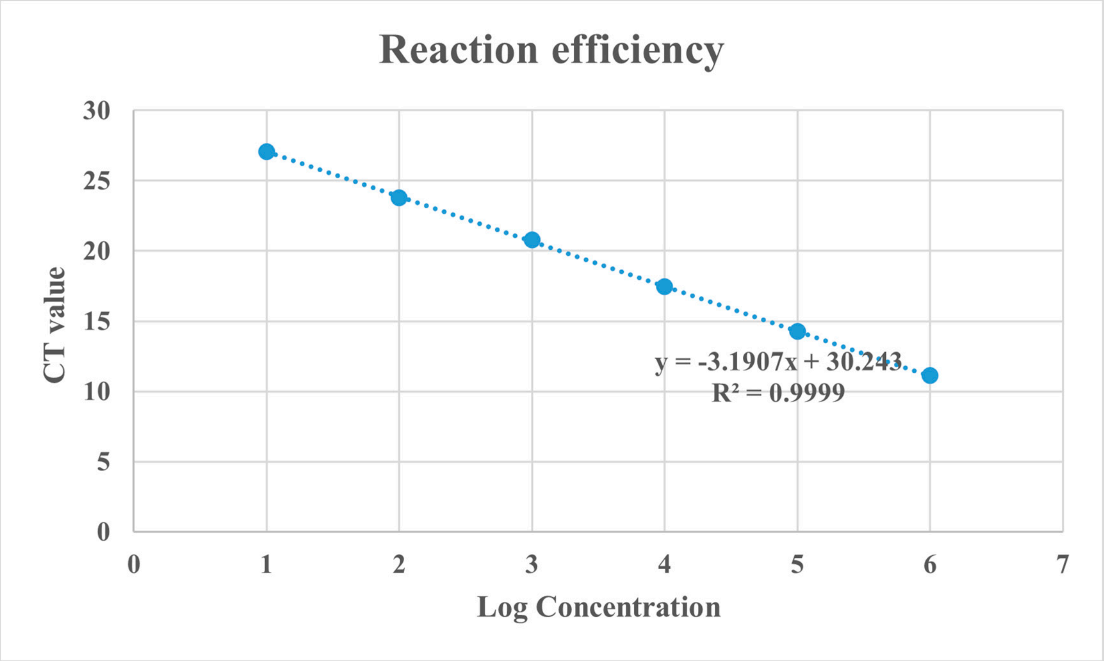

Figure S1. Standard curves for reaction efficiency of q-PCR.
